# Molecular Authentication of Twelve Meat Species Through a Promising Two-Tube Hexaplex Polymerase Chain Reaction Technique

**DOI:** 10.3389/fnut.2022.813962

**Published:** 2022-03-24

**Authors:** Zhendong Cai, Guowei Zhong, Qianqian Liu, Xingqiao Yang, Xiaoxia Zhang, Song Zhou, Xiaoqun Zeng, Zhen Wu, Daodong Pan

**Affiliations:** ^1^Key Laboratory of Animal Protein Deep Processing Technology of Zhejiang Province, College of Food and Pharmaceutical Sciences, Ningbo University, Ningbo, China; ^2^Center for Global Health, School of Public Health, Nanjing Medical University, Nanjing, China; ^3^Institute of Environmental Research at Greater Bay Area, Guangzhou University, Guangzhou, China; ^4^Ordos Agriculture and Animal Husbandry Technology Extension Centre, Ordos, China

**Keywords:** molecular authentication, hexaplex PCR, meat adulteration, species-specific primer, commercial foodstuffs

## Abstract

Frequent meat frauds have aroused significant social attention. The aim of this study is to construct a two-tube hexaplex polymerase chain reaction (PCR) method offering accurate molecular authentication of twelve meat species in actual adulteration event. Deoxyribonucleic acid (DNA) sequencing demonstrates that designed primers can specifically amplify target species from genomic DNA mixture of six species in each tube reaction, which showed 100% accuracy of horse (148 bp), pigeon (218 bp), camel (283 bp), rabbit (370 bp), ostrich (536 bp), and beef (610 bp) as well as turkey (124 bp), dog (149 bp), chicken (196 bp), duck (277 bp), cat (380 bp), and goose (468 bp). A species-specific primer pair produced the target band in the presence of target genomic DNA but not non-target species. Through multiplex PCR assays with serial concentration of the DNA mixture of six species in each PCR reaction, the detection limit (LOD) of the two-tube hexaplex PCR assay reached up to 0.05–0.1 ng. Using genomic DNA isolated from both boiled and microwave-cooked meat as templates, PCR amplification generated expected PCR products. These findings demonstrate that the proposed method is specific, sensitive and reproducible, and is adequate for food inspection. Most importantly, this method was successfully applied to detect meat frauds in commercial meat products. Therefore, this method is of great importance with a good application foreground.

## Introduction

Meat products contribute essential nutrients to human such as proteins, fatty acids, trace elements, and vitamins, especially for the richest protein source (1). Based on the growing demand for animal protein-based foods, meat frauds such as counterfeiting and mislabeling have become a severe global issue (2–4). Over the last two decades, events of meat adulteration have occurred globally because of pursuit of extra economic benefits (2). The notorious horsemeat scandal in the European Union in 2013 is a high-profile food fraud incident, which has shaken consumer trust in food industries throughout the globe (5). Besides, meat adulteration not only breaks market rules but also violates ethical norms and religious laws. As known, pork has strictly restricted consumption in Islam and Judaism, and beef is prohibited in Hindus (6, 7). In addition, meat fraud risks food safety and even threatens public health because of metabolic disorders, allergies, or infectious diseases. As reported, forbidden ingredients such as fox and mouse are occasionally mixed into edible meat products (3, 8). Nevertheless, allergic reactions can be triggered by some meat species especially for sensitized patients (9). Hence, authentication of meat products is necessary to protect consumers from meat fraud and ensure public health in dietary practices.

A reliable, sensitive, and low-cost analytical technique is of great importance to ensure food quality and protect consumers from being deceived. Techniques have been greatly improved recently due because of progress in molecular biology. Deoxyribonucleic acid (DNA) molecules are present in cells and possess excellent stability under high temperature and pressure and chemical processing, suggesting that DNA-based analytical methods are reliable for detection of meat frauds. Polymerase chain reaction (PCR) techniques such as species-specific PCR, multiplex PCR, PCR-RFLP, real-time PCR, random amplified polymorphic DNA (RAPD), and DNA barcoding have evolved as preferred methods for meat fraud detection (2, 10, 11). Recently, both multiplex and real-time PCR techniques have been widely applied for meat fraud detection (12). Real-time PCR provides more detailed information regarding the identification and quantification of meat species (13). However, the matrix may interfere with the amplification process, such that accurate quantification could only be achieved in the presence of a proper reference material (13), indicating that it is difficult to quantify meat fractions in real-world foodstuffs. In addition, real-time PCR assays depend on suitable equipment and trained professionals. In contrast, multiplex PCR is a particularly desirable method, which can efficiently authenticate more species and visually observed through simple agarose gel analysis, suggesting that multiplex PCR assays can be easily implemented with minimum effort but much gain to verify the identification of meat species.

Mitochondrial DNA (mtDNA) harboring multiple copies in all cells possess intraspecies conservation and interspecies polymorphism, so mitochondrial genes are preferred targets for meat fraud detection. Here, using mtDNA genes including NADH dehydrogenase subunits 5 and 6, 12S and 16S rRNAs, cytochrome c oxidase subunits I and II, D-loop, and cytochrome *b* as targets, species-specific primers for twelve animal species (horse, pigeon, camel, rabbit, ostrich, beef, turkey, dog, chicken, duck, cat, and goose) are designed and screened based on tests of cross-reactivity, specificity, sensitivity, and robustness. A two-tube hexaplex PCR assay, which efficiently detects twelve animal origins, is ultimately developed with twelve pairs of species-specific primers. Moreover, this method is adequate for assessment of fraud incidences in commercial meat products.

## Materials and Methods

### Sample Collection and Deoxyribonucleic Acid Extraction

According to a previous approach (14), fresh meat samples of the twelve animal species were purchased from local retailers and markets in Ningbo City, People’s Republic of China. Commercial samples in triplicates were purchased on different dates from local supermarkets and online supermarket platforms. All the samples were transported under ice-chilled state and were stored at −80°C to inhibit DNA degradation until further use for DNA extraction. Genomic DNA was isolated by using the EasyPure^®^ Genomic DNA Kit (TransGen Biotech Co., Ltd., Beijing, China) according to the manufacturer’s instructions. DNA concentration was measured with a NanoDrop 2000 UV-Vis spectrophotometer (Thermo Scientific, Wilmington, NC, United States).

### Design of Species-Specific Primers

Because of high divergence and conservation of mitochondrial sequences within the animal species, mitochondrial genes were selected as targets for designing primers (15). The mitochondrial genes shown in Table 1 are retrieved from the National Center of Biotechnology Information (NCBI) database. Combining the Oligo 7.0 and BLAST programs, species-specific primers were designed based on physical parameters of cross-reactivity, melting temperature, self-complementarity, and secondary structures. To check *in silico* specificity, the primers were aligned against target and non-target animal species, including 14 land animals of horse (*Equus caballus*), pigeon (*Columba livia*), camel (*Camelus bactrianus*), rabbit (*Oryctolagus cuniculus*), ostrich (*Struthio camelus*), cattle (*Bos taurus*), turkey (*Meleagris gallopavo*), dog (*Canis lupus*), chicken (*Gallus gallus*), duck (*Anas platyrhynchos*), cat (*Felis catus*), goose (*Anser cygnoides*), and pig (*Sus scrofa*), sheep (*Ovis aries*), and 3 aquatic species of small yellow croaker (*Larimichthys polyactis*), tuna (*Thunnus orientalis*), and black carp (*Mylopharyngodon piceus*) using a ClustalW sequence alignment program and the MEGA6 software. Finally, a cross-amplifcation reaction was individually examined to validate the species-specificity of primer pairs by simplex PCR assays. Optimized sequences of primer sets in detail are shown in Table 1.

**TABLE 1:** Oligonucleotide primers for the meat species used in this study.

Primers	Genes	Sequence (5′-3′ direction)	Amplicons (bp)	Reference or source
Horse	NADH dehydrogenase subunit 5	CCCCGCTTCCTCCCTCTGA	148	This study
		TAGGTATGGTTATTTCCGGGACG		
Pigeon	NADH dehydrogenase subunit 5	GGCCCAGAAAGCATCACCTC	218	This study
		ATTGGTATAGCGATTAGGGACAG		
Camel	16S rRNA	CTAGCCCAGAAAATACCACAT	283	This study
		CATAGACGAGTTCGCTCCGTA		
Rabbit	NADH dehydrogenase subunit 5	AATCCGCTTCTACCCCTTG	370	This study
		TATACCTGTGAGGGCCAGACT		
Ostrich	16S rRNA	AGCGCCCTCTAGCTCATCC	536	This study
		GCTGCTTTAGGGCCAACGTG		
Beef	Cytochrome c oxidase subunit I	ATGAGCCCACCATATATTCACT	610	This study
		TGTCGTGGTTAAGTCTACAGTCA		
Turkey	Cytochrome c oxidase subunit II	AGTTGACCACCGTATAGTAGTCC	124	This study
		TCGTCCTGGGATTGCATCTGTCT		
Dog	D-loop	CCCTTGCTCGTAATGTCCCT	149	This study
		CGAGATGTCCCATTTGCGAGA		
Chicken	12S rRNA	CAGGTATCAGGCACACTCAGC	196	This study
		CACTCTTTACGCCGGGTAGC		
Duck	NADH dehydrogenase subunit 6	CCACGCGAATAAAGCATAGCC	277	This study
		TTTCGTTTGTAGCCCTGGTG		
Cat	Cytochrome c oxidase subunit I	TCTTAGCAGCGGGAATCACT	380	This study
		AAGAGTAGCCAGTCAACTAAACA		
Goose	Cytochrome b	TCGCCTTCTCCTCAGTAGCTC	468	This study
		TGTCGCAGTCTGATACGATT		
Eukaryotes	12S rRNA	CAACTGGGATTAGATACCCCACTAT	456	(24)
		GAGGGTGACGGGCGGTGTGT		
Eukaryotes	16S rRNA	AAGACGAGAAGACCCTATGGA	240	(25)
		GATTGCGCTGTTATCCCTAGGGTA		
Eukaryotes	18S rRNA	AGGATCCATTGGAGGGCAAGT	99	(26)
		TCCAACTACGAGCTTTTTAACTGCA		

### Simplex and Multiplex Polymerase Chain Reaction Assays

Polymerase chain reaction assays were performed as previously described (14). Simplex PCR for each species with its own primers was carried out using an EasyTaq^®^ DNA Polymerase kit (TransGen Biotech Co., Ltd., Beijing, China). The PCR reaction system included 2.5 μl EasyTaq^®^ Buffer (10 x), 2 μl dNTPs (2.5 mM), 0.5 μl EasyTaq DNA Polymerase (5 units μl^–1^),0.5 μl each primer (10 μM), 1 μl genomic DNA (1 ng μl^–1^), and refilled ddH_2_O to 25 μl. PCR reaction was initiated by 5-min denaturation at 94°C, followed by 34 cycles of 94°C for 30 s, 63°C for 30 s, 72°C for 45 s, and final elongation at 72°C for 5 min. For multiplex PCR, the PCR reaction system included 2.5 μl EasyTaq^®^ Buffer (10 x), 2 μl dNTPs (2.5 mM),0.5–1 μl EasyTaq DNA Polymerase (5 units μl^–1^),0.5 μl each primer of all six species (10 μM),1 μl genomic DNA of each species at indicated concentrations from 10 to 0.1 ng μl^–1^, and refilled ddH_2_O to 25 μl. Using the same PCR amplification condition as that of simplex PCR, a two-tube hexaplex PCR assay was developed using two sets of six species-specific primer pairs and corresponding DNA mixture of six species in two tubes. All PCR fragments were amplified using T100 Thermal Cycler (Bio-Rad, Germany). PCR products were loaded into 4% agarose gel using 4S GelRed Nucleic Acid Stain and were visualized by Gel Doc XR + System with Image Lab Software (BIO-RAD) (16).

### Sequencing of Polymerase Chain Reaction Products

Deoxyribonucleic acid (DNA) sequencing was performed as previously described with some modifications (17). The PCR product was isolated, purified, and then cloned into a *pEASY*^®^-T5 zero cloning vector (TransGen Biotech Co., Ltd., Beijing, China). PCR amplification with vector primers M13F and M13R was carried out using the template of plasmid DNA and then sequenced. Sequencing was accomplished with an automated DNA sequencer (Applied Biosystems, Foster City, CA, United States). The DNA base composition of the sequence was determined by a BLAST search against the NCBI nucleotide database.

### Specificity, Sensitivity, and Reproducibility of the Primers

The specificity of each primer pair was assessed using template DNA extracted from the twelve species by simplex and multiplex PCR assays. The results were run on 4% agarose gel and then visualized for proper amplification. The sensitivity of multiplex PCR was determined by g serial dilutions of the premixed genomic DNA templates of six target species in each tube reaction. Ten concentrations ranging from 10 to 0.01 ng and species-specific primers of six species were used for PCR amplification. The limit of detection and dynamic range were analyzed through 4% agarose gel and electropherograms. For the reproducibility test, meat samples of all the species were boiled at 97–99°C for 30 min. Other meat samples of all the species were, respectively microwave-cooked at 750 W for 10 min. After both heat processing treatments, genomic DNA of each species was extracted and used for PCR amplification to examine reproducibility (17).

### Commercial Samples

A total of 55 samples including raw and heat processing of meat balls (5), meat slices (10), kebab (5), sausages (5), jerky (14), drysalter (6), dry meat stripe (2), cutlets (5), and sauce braised meat (3) were purchased from markets as well as online supermarket platforms. Genomic DNA of each sample was isolated and used as the template for meat authentication using the proposed hexaplex PCR method. Detailed information of the samples is listed in Table 2.

**TABLE 2:** Results of multiplex PCR assay performed on commercial meat products.

Products (number)	Detected species											
	Horse	Pigeon	Camel	Rabbit	Ostrich	Beef	Turkey	Dog	Chicken	Duck	Cat	Goose
**Beef (15)**												
Meat balls (5)						5/5			1/5^a^, 1/5^b^	1/5^b^		
Meat slices (5)						5/5			1/5^a^	1/5^b^		
Kebab (5)	1/5^a^				1/5^b^	5/5						
**Horse (10)**												
Meat slices (2)	2/2		1/2^a^									
Sausages (5)	5/5								1/5^a^	1/5^a^, 1/5^b^		
Jerky (3)	3/3											
**Camel (10)**												
Drysaltery (3)			3/3		1/3^a^							
Dry meat stripe (2)			2/2									
Jerky (5)	1/5^a^		5/5									
**Ostrich (10)**												
Drysaltery (3)					3/3		1/3^a^					
Jerky (4)		1/4^b^			4/4				1/4^a^			
Sauce braised meat (3)					3/3							
**Turkey (10)**												
Cutlets (5)							5/5		1/5^a^			1/5^b^
Meat slices (3)							3/3		1/3^a^			
Jerky (2)							2/2					

*In each row, the meat samples labeled with same letter (a or b) represent the identical meat samples, while different letters indicate a difference in meat samples.*

## Results

### Specificity of Polymerase Chain Reaction Assay

To obtain species-specific primers for horse, pigeon, camel, rabbit, ostrich, beef, turkey, dog, chicken, duck, cat, and goose, candidate primers for each of the animal species were designed using the Oligo 7.0 and BLAST programs. First, the specificity of each species-specific primer pair was individually checked by simplex PCR assays through cross-amplification reaction with 16 non-target species including all the twelve species indicated, and all of which showed no cross-reactivity (data not shown). PCR amplification showed distinct bands with a predicted size of 148 bp, 218 bp, 283 bp, 370 bp, 536 bp, and 610 bp for the horse, pigeon, camel, rabbit, ostrich, and beef species, respectively (Supplementary Figure 1A). As positive controls, three universal eukaryotic primer pairs, which target 18S rRNA, 16S rRNA, and 12S rRNA genes, were employed in one tube PCR reaction. As seen in Supplementary Figure 1B, all the meat samples generated a predicted size of 99 bp, 240 bp, and 456 bp with similar intensities, suggesting good quality of template DNAs of each meat resource. In addition, using genomic DNA of single species as the template, the target band could be produced in the presence of a complete mixture of six primer pairs but not five non-target primer pairs (Supplementary Figure 1C). Likewise, a species-specific primer pair produced the target band in the presence of DNA mixture of six meat species but not five non-target species (Supplementary Figure 1D).

The primer specificity for turkey, dog, chicken, duck, cat, and goose was also investigated. As shown in Supplementary Figure 2A, the PCR amplification shows the predicted bands for turkey (124 bp), dog (149 bp), chicken (196 bp), duck (277 bp), cat (380 bp), and goose (468 bp). Using three universal eukaryotic primer pairs, the PCR assay demonstrated that good quality of genomic DNA was present in all six meat samples, ensuring the accuracy of the experiment (Supplementary Figure 2B). Using the genomic DNA of single species as the template, PCR amplification generated the target band in the presence of a complete mixture of six primer pairs but not five non-target ones (Supplementary Figure 2C). PCR amplification with each species-specific primer pair generated the target band in the presence of the template DNA mixture of six meat species but not five non-target species (Supplementary Figure 2D).

To further confirm the accuracy of PCR amplification, amplicons for all the species in Supplementary Figures 1D, 2D are individually cloned and sequenced. Target species with 100% accuracy was verified by a BLAST search against the NCBI nucleotide database. Partial data of DNA sequencing for each target species are shown in Supplementary Figures 3A,B. Collectively, all the experiments conclude that the designed primers are highly specific and are adequate for food inspection.

### Sensitivity of Multiplex Polymerase Chain Reaction Assay

**FIGURE 1:**
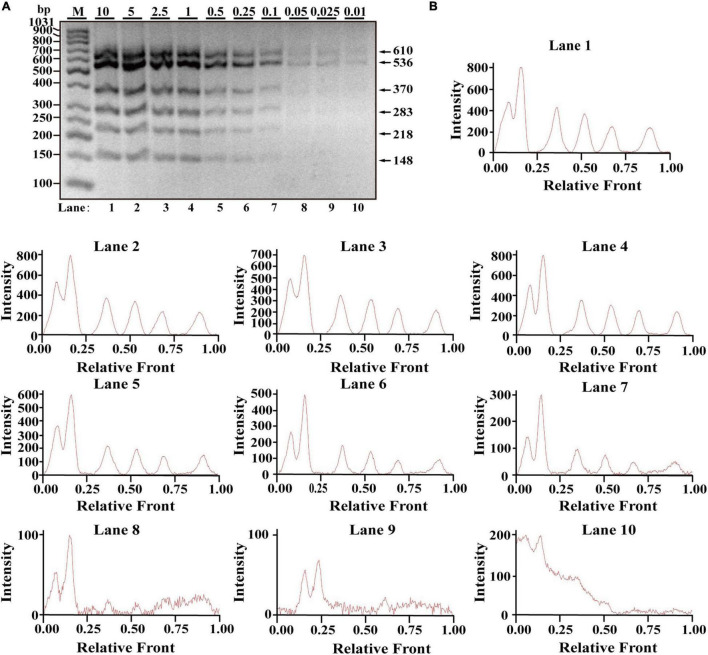
Validation of the sensitivity of the one-tube hexaplex PCR assay. **(A)** Gel image of multiplex PCR fragments amplified with six primer pairs and DNA mixture of six species: horse, pigeon, camel, rabbit, ostrich, and beef under the indicated concentration in a single PCR reaction. **(B)** Using Image Lab Software, electropherograms were drawn based on bands. Lanes 1–10 are represented labels of 10, 5, 2.5, 1, 0.5, 0.25, 0.1, 0.05, 0.025, and 0.01 in **(A)**. The value of number in the horizontal line means the relative position of peaks distant from the top of agarose gel. The value of number at the vertical line means the fluorescent intensity of PCR fragments using 4S GelRed Nucleic Acid Stain. Lane M is ladder DNA.

**FIGURE 2:**
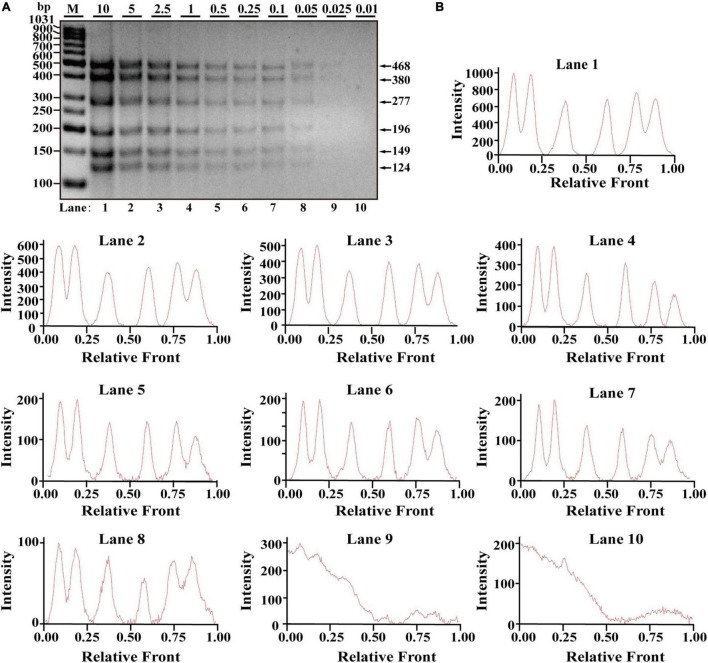
Validation of the sensitivity of the one-tube hexaplex PCR assay. **(A)** Gel image of multiplex PCR fragments amplified with six primer pairs and DNA mixture of six species: turkey, dog, chicken, duck, cat, and goose under the indicated concentration in a single PCR reaction. **(B)** Using Image Lab Software, electropherograms were drawn based on bands. Lanes 1–10 are represented labels of 10, 5, 2.5, 1, 0.5, 0.25, 0.1, 0.05, 0.025, and 0.01 in **(A)**. The value of number in the horizontal line means the relative position of peaks distant from the top of agarose gel. The value of number at the vertical line means the fluorescent intensity of PCR fragments using 4S GelRed Nucleic Acid Stain. Lane M is ladder DNA.

After optimization of simplex PCR for individual species, multiplex PCRs starting from duplex, triplex, tetraplex, and pentaplex were attempted to be constructed, and a two-tube hexaplex PCR assay was ultimately developed using six pairs of species-specific primers in each tube. To reveal the limit of detection (LOD) and dynamic range of the two hexaplex PCR assays, PCR assays were performed with the serial concentration of each DNA template ranging from 10 to 0.01 ng per PCR reaction. Visible bands were matched with intact peak patterns, while weak bands were equipped with defective peak patterns. As template DNA amounts of each species were less than 0.1 ng, PCR fragments were almost invisible for the horse, pigeon, camel, rabbit, ostrich, and beef species in one tube reaction (Figure 1A). On the whole, fluorescence intensities were gradually decreased by reducing the content of genomic DNA template, reflecting their reduced PCR products. With decreasing fluorescence intensity, there were six visible peak patterns in lines 1–7 but not lines 8–10 (Figure 1B), suggesting that the threshold value of genomic DNA was about 0.1 ng. Therefore, the LOD of hexaplex PCR method for the horse, pigeon, camel, rabbit, ostrich, and beef species was approximately 0.1 ng in one tube reaction. Likewise, it was concluded that the detection threshold of the hexaplex PCR method for turkey, dog, chicken, duck, cat, and goose in the other tube reaction was about 0.05 ng template DNA, which has six bands as shown in Figure 2A and six peaks as shown in Figure 2B.

### Reproducibility of Polymerase Chain Reaction Assay in Heat-Processed Meat

To determine the availability of primers for detecting animal origin in thermally processed meat, both boiled and microwave-cooked treatments were selected to process raw meat tissues. The genomic DNA of each species was isolated from heat processed meat tissues. PCR amplification generated the expected PCR products with 100% accuracy as that of raw meat samples in heat processing animal species of horse, pigeon, camel, rabbit, ostrich and beef, respectively (Supplementary Figures 4A,B). Similar results are obtained from PCR amplification of the turkey, dog, chicken, duck, cat, and goose species, as shown in Supplementary Figures 4C,D. Taken together, the results suggest that the designed primers are qualified for detecting meat ingredients in real-world meat products.

### Application of Multiplex Polymerase Chain Reaction Assay in Commercial Meat Products

Since some food items such as meatballs, meat slices, kebab, drysalter, and jerky are highly popular and have high consumption rate, 55 commercial samples were randomly selected for multiplex PCR analysis. As summarized in Table 2, most of the meat samples declared to be having 100% pure meat content contained the identical ingredients as labeled. However, some shocking findings that samples were adulterated with extra ingredients were unmasked. As illustrated, 6 of 15 (40%) beef samples, 3 of 10 (30%) horse samples, 2 of 10 (20%) camel samples, 3 of 10 (30%) ostrich samples, and 3 of 10 (30%) turkey samples contained meat ingredients that were unlisted. From this survey, the incidence of meat frauds is still rampant until now, especially for some kinds of poultry meat that are fraudulently mixed or counterfeited with red meat. The survey further corroborates the availability of this two-tube hexaplex PCR assay in authenticating commonly consumed meat ingredients.

## Discussion

The multiplex PCR technique is a highly effective method for detecting multiple targets in a single platform, which dramatically cuts the cost and time of analysis through a simple agarose gel analysis (18–20). Notably, with the increase of primers and multiplicity of PCR reaction, mutual interference of PCR components causes lower efficiency and even the failure of amplification (7). Through analyses of some bodies of literature recently published, multiplex PCRs are summarized and shown in Table 3. Multiplex PCRs such as duplex, triplex, tetraplex, pentaplex (quintuple), and hexaplex (sextuple) have been broadly reported for meat authentication, while most multiplex PCR assays have authenticated less than eight meat species. To our knowledge, relatively little is known about multiplex PCRs that discriminate more than ten animal species. Although two studies have authenticated ten and fourteen animal species, they are achieved by two-tube multiplex PCR assays (7, 17). Notably, fourteen animal species were detected by two-tube independent pentaplex PCR assays with ten pairs of primers, three of them used degenerate primers (7). However, the degenerate primers inaccurately distinguish sheep and goat in ovis, dog, fox, and raccoon-dog in Canidae, and chicken and duck in poultry (7). Our previous study has developed a septuple PCR assay for identifying seven species of turkey, goose, pig, sheep, beef, chicken, and duck in one tube reaction, but it fails to simultaneously authenticate more than seven species (14). In this research, multiplex PCRs have also been found to confront technological challenge, because multiplex PCR with increasing species-specific primers in one reaction sometimes generates loss or unexpected bands. Through screening new species-specific primers and optimizing species combination, a two-tube hexaplex PCR method was ultimately established for accurate authentication of twelve meat species.

**TABLE 3:** Comparative analysis of multiplex PCR assays for the identification of meat species.

Multiplex PCR type	Species number	Detection items	Detection limit	Detection method	References or source
Multiplex (two-tube)	12	Horse, pigeon, camel, rabbit, ostrich, beef; turkey, dog, chicken, duck, cat, and goose	0.05–0.1 ng DNA	Gel	This study
Multiplex (two-tube)	14	Cattle, donkey, canidae (dog, fox, raccoon-dog), deer, horse; pig, ovis (sheep, goat), poultry (chicken, duck), cat, and mouse	0.02–0.2 ng DNA	Chip	(7)
Multiplex (two-tube)	10	Beef, sheep, pork, chicken, turkey; cat, dog, mouse, rat, and human	30 pg DNA	Gel	(17)
Octuplex	8	Dog, chicken, cattle, pig, horse, donkey, fox, and rabbit	0.05 ng/μL DNA	Gel	(25)
Heptaplex (RFLP)	7	Beef, buffalo, chicken, duck, goat, sheep, and pork	0.5% for each species	Chip	(4)
Septuple PCR	7	Turkey, goose, pig, sheep, beef, chicken, and duck	0.01–0.05 ng DNA	Gel	(14)
Multiplex	6	Mutton, pork, duck, chicken, horse, cat	9.1% of each species	Gel	(27)
Multiplex	6	Goat, chicken, cattle, sheep, pig, horse	0.25 ng	Gel	(28)
Multiplex	5	Sheep/goat, bovine, chicken, duck, and pig	0.5 ng	Gel	(19)
Pentaplex	5	Dog, duck, buffalo, goat, sheep	0.1–0.32 ng DNA	Gel	(13)
Quadruplex	4	Chicken, mutton, beef, pork	16 pg DNA, 0.01% of each species	Gel	(29)
Quadruple	4	Fox, mink, or raccoon in beef, and mutton	1% for each species	Gel	(30)
Multiplex	4	Buffalo, cattle, pork, and duck	1 pg DNA, 0.1% for each species	Gel	(31)
Quadruple	4	Beef, pork, mutton, and duck	0.1 ng DNA	Gel	(32)
Tetraplex	3	Pig, cattle, and fish	0.001–0.1 ng DNA	Gel	(33)
Multiplex	3	Chicken, duck, and goose	0.05 ng DNA or 1% for each species	Gel	(34)
Multiplex	2	Cattle, horse	0.05 ng DNA	Gel	(3)
Multiplex	2	Cattle, buffalo	2.23–2.31 ng/μL DNA	Gel	(35)

*Chip, microchip electrophoresis; Gel, agarose gel electrophoresis.*

Nevertheless, multiplex PCR occasionally causes artifacts because of contamination by alien DNA even at a very low level and generates non-specific target amplification (21). To eliminate the possibility, each species-specific primer pair was individually used for amplifying target species using the DNA mixture of all six species as a template in one PCR reaction. PCR products were subsequently connected into a cloning vector for DNA sequencing, which is highly promising and reliable for determination of nucleotide base sequences. BLAST analysis confirmed species-specific PCR amplification for all the species with 100% accuracy, suggesting that the developed system can accurately amplify each target. Application of the two-tube hexaplex PCR assay in commercial meat products further validated the availability of the developed system. In accordance with other reports, meat fraud with cheap or poor-quality meat has become common worldwide (4, 7). Based on this, accurate verification of meat ingredients is crucial to safeguard consumers from meat fraud and thereby contributes to establish discipline in food business.

According to the data shown in Table 2, the proposed method reveals the phenomenon that commercial meat products are frequently adulterated. However, they showed morphological and physical features similar to that of pure meat, indicating that the practice of meat adulteration has been ingeniously performed. Therefore, a reliable analytical technique with high sensitivity is required for meat authentication. In this study, the LOD of the two-tube hexaplex PCR assay reached up to0.05–0.1 ng. Compared with the LOD of multiplex PCR assays shown in Table 3, the developed technique is qualified for discriminating meat source. Molecular authentication or molecular traceability of meat species, which is based on the developed multiplex PCR amplification of genomic DNA, has provided an accurate analysis of meat ingredients (22, 23). In this regard, the proposed PCR method targeting mtDNA to authenticate twelve animal species in food products would be especially useful.

## Conclusion

This study provides a two-tube independent hexaplex PCR assay for molecular authentication of meat fraud, which is a reliable, low-cost, and rapid approach, and offers unambiguous detection and discrimination of twelve animal species. Furthermore, the technique has been corroborated for its accuracy, specificity, sensitivity, and applicability in commercial meat products. The proposed method is of great importance and will have a good application foreground.

## Data Availability Statement

The original contributions presented in the study are included in the article/Supplementary Material, further inquiries can be directed to the corresponding authors.

## Author Contributions

ZC, QL, and DP: conception and design of the investigation and study. QL, GZ, XY, XxZ, SZ, XqZ, ZW, and ZC: completion of the experiments. QL, GZ, and ZC: evaluation and analysis of the results. QL, GZ, ZC, and DP: manuscript writing. QL, GZ, XY, XxZ, SZ, XqZ, ZW, ZC, and DP: final approval of the manuscript. All authors contributed to the article and approved the submitted version.

## Conflict of Interest

The authors declare that the research was conducted in the absence of any commercial or financial relationships that could be construed as a potential conflict of interest.

## Publisher’s Note

All claims expressed in this article are solely those of the authors and do not necessarily represent those of their affiliated organizations, or those of the publisher, the editors and the reviewers. Any product that may be evaluated in this article, or claim that may be made by its manufacturer, is not guaranteed or endorsed by the publisher.
