# Supplementary material for: Molecular Authentication of Twelve Meat Species Through a Promising Two-Tube Hexaplex Polymerase Chain Reaction Technique
Source: Front Nutr. 2022 Mar 24;9:813962. doi: 10.3389/fnut.2022.813962 (PMC8989424; doi:10.3389/fnut.2022.813962)
Supplement: Supplementary file 1 [file Presentation_1.pdf]

**Molecular authentication of twelve meat species through a promising two-tube hexaplex PCR technique**

Zhendong Cai[1,†], Guowei Zhong[2,†], Qianqian Liu[3,*], Xingqiao Yang[1], Xiaoxia Zhang[4], Song Zhou[1], Xiaoqun Zeng[1], Zhen Wu[1], Daodong Pan[1,*]

***Supplementary Material***

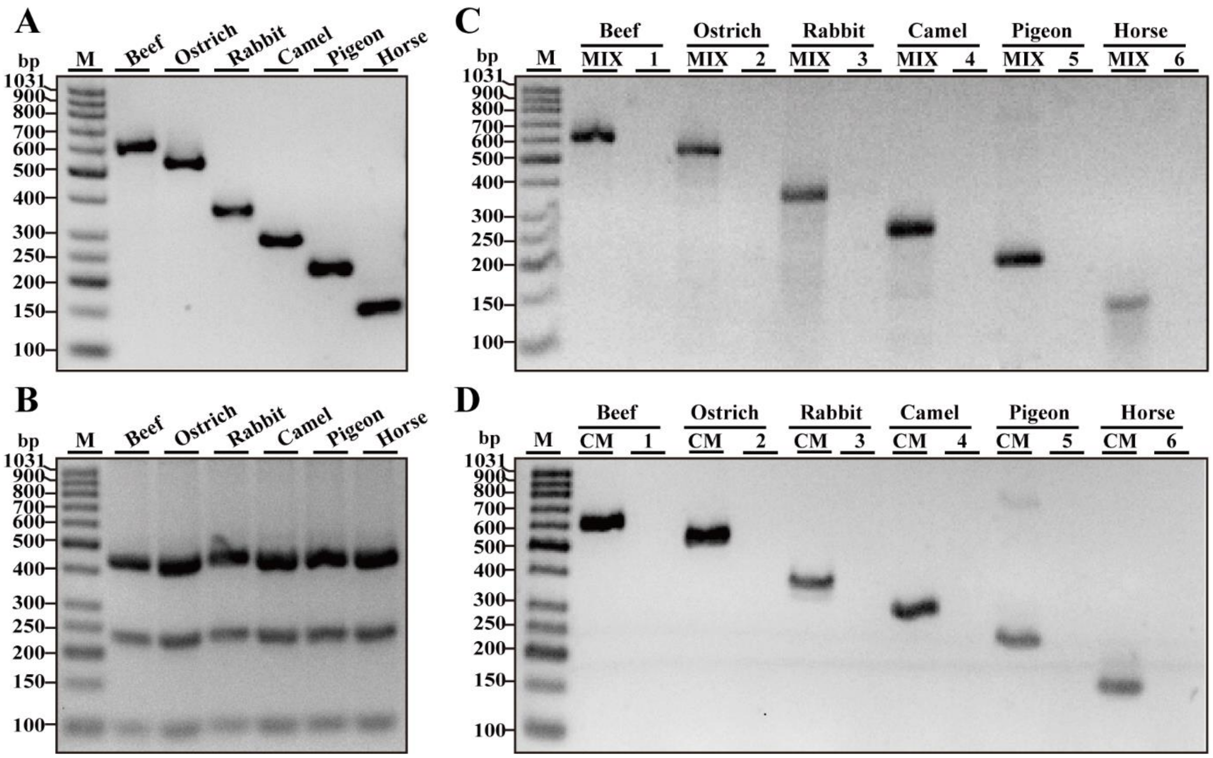


**Supplementary Figure 1.** Verification of the primer specificity. (A) PCR fragments amplified with species-specific primers for horse, pigeon, camel, rabbit, ostrich and beef using respective genomic DNA as a template. (B) PCR fragments amplified with premixed universal primers for each meat sample. (C) PCR amplification with premixed primers indicated and genomic DNA of a single meat species as the template. MIX, a mixture of six primer pairs in one tube reaction; 1–6, a mixture of five primer pairs for five nontarget species. (D) PCR amplification with each species-specific primer pair and DNA mixture of meat species indicated. CM, a complete DNA mixture of six meat species; 1–6, DNA mixture of five meat species except target species. Lane M is ladder DNA.

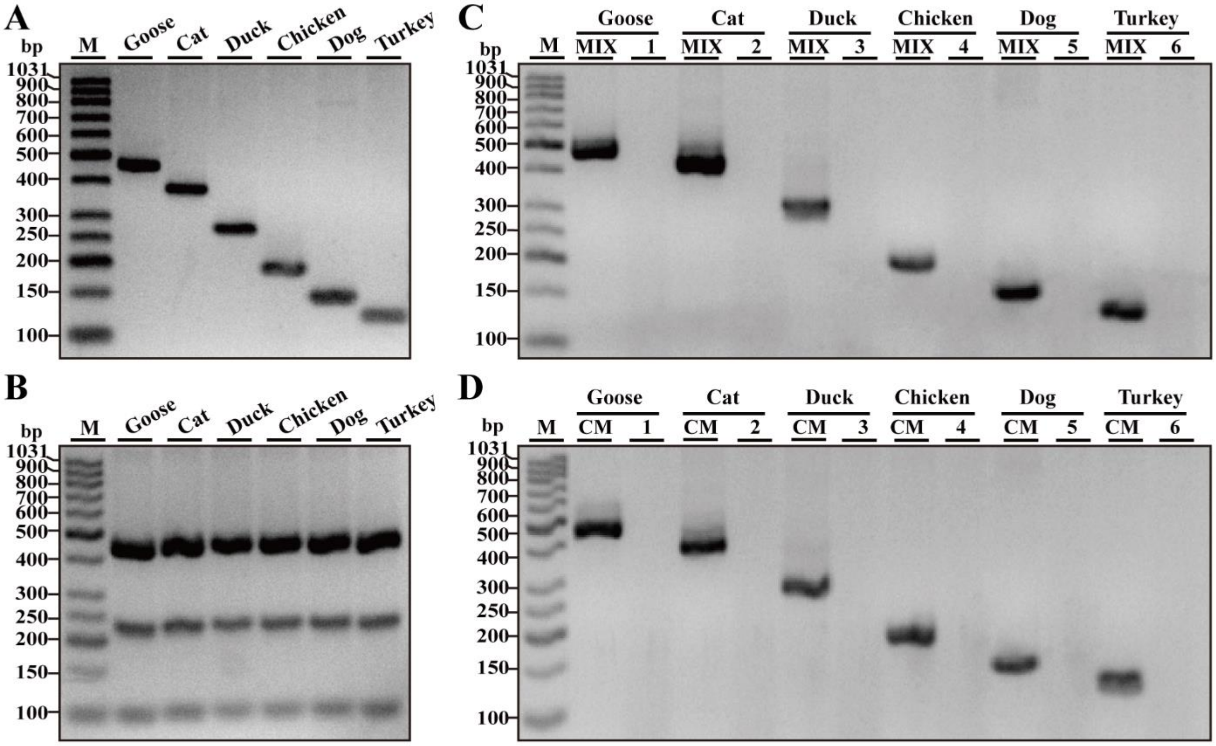


**Supplementary Figure 2.** Verification of the primer specificity. (A) PCR fragments amplified with species-specific primers for turkey, dog, chicken, duck, cat and goose using respective genomic DNA as a template. (B) PCR fragments amplified with premixed universal primers for each meat species indicated. (C) PCR amplification with premixed primers indicated and genomic DNA of a single meat species as a template. MIX, a mixture of six primer pairs for six animal species indicated in one tube reaction; 1–6, a mixture of five primer pairs for five nontarget species. (D) PCR amplification with each species-specific primer pair and DNA mixture of meat species indicated. CM, a complete DNA mixture of six meat species; 1–6, DNA mixture of five meat species except target species.

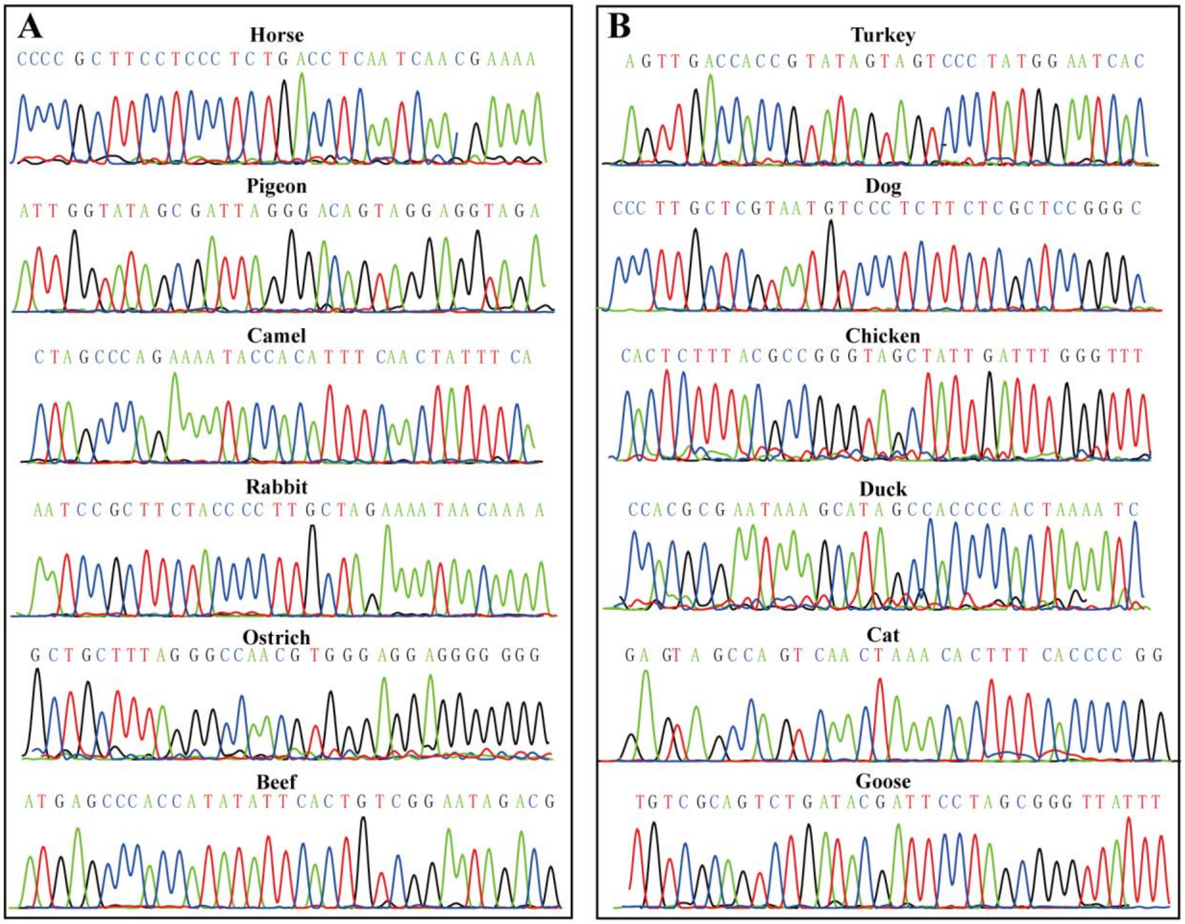


**Supplementary Figure 3.** (A) Partial results of DNA sequencing of PCR fragments amplified from horse, pigeon, camel, rabbit, ostrich and beef species, respectively. (B) Partial results of DNA sequencing of PCR fragments amplified from turkey, dog, chicken, duck, cat and goose species, respectively.

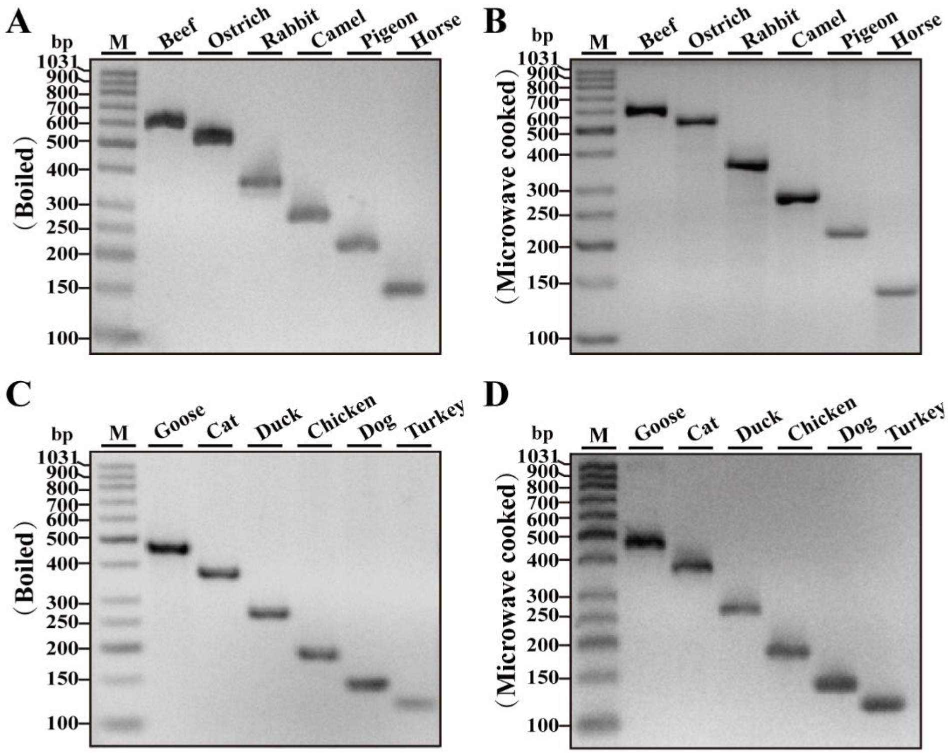


**Supplementary Figure 4.** Validation of the availability of species-specific primers in heat processing meat. Genomic DNA was respectively extracted from boiled (A) and microwave-cooked (B) meat samples of horse, pigeon, camel, rabbit, ostrich and beef. Genomic DNA was respectively extracted from boiled (C) and microwave-cooked (D) meat samples of turkey, dog, chicken, duck, cat and goose. PCR fragments were amplified with species-specific primer pair and individual genomic DNA as a template. Lane M is ladder DNA.
